# Differential Epigenetic Changes in the Dorsal Hippocampus of Male and Female SAMP8 Mice: A Preliminary Study

**DOI:** 10.3390/ijms241713084

**Published:** 2023-08-23

**Authors:** Federico Ravanelli, Laura Musazzi, Silvia Stella Barbieri, Gianenrico Rovati, Maurizio Popoli, Alessandro Barbon, Alessandro Ieraci

**Affiliations:** 1 Department of Pharmaceutical Sciences, University of Milan, 20133 Milan, Italy; federico.ravanelli@studenti.unimi.it (F.R.); genrico.rovati@unimi.it (G.R.); maurizio.popoli@unimi.it (M.P.); 2 Department of Medicine and Surgery, University of Milano-Bicocca, 20900 Monza, Italy; laura.musazzi@unimib.it; 3 Unit of Brain-Heart Axis: Cellular and Molecular Mechanisms, Centro Cardiologico Monzino IRCCS, 20138 Milan, Italy; silvia.barbieri@cardiologicomonzino.it; 4 Department of Molecular and Translational Medicine, University of Brescia, 25123 Brescia, Italy; alessandro.barbon@unibs.it; 5 Department of Theoretical and Applied Sciences, eCampus University, 22060 Novedrate, Italy

**Keywords:** aging, Alzheimer’s disease, SAMP8 mice, histone post-translational modifications, sex difference

## Abstract

Alzheimer’s disease (AD) is the most common age-related neurodegenerative disease characterized by memory loss and cognitive impairment. The causes of the disease are not well understood, as it involves a complex interaction between genetic, environmental, and epigenetic factors. SAMP8 mice have been proposed as a model for studying late-onset AD, since they show age-related learning and memory deficits as well as several features of AD pathogenesis. Epigenetic changes have been described in SAMP8 mice, although sex differences have never been evaluated. Here we used western blot and qPCR analyses to investigate whether epigenetic markers are differentially altered in the dorsal hippocampus, a region important for the regulation of learning and memory, of 9-month-old male and female SAMP8 mice. We found that H3Ac was selectively reduced in male SAMP8 mice compared to male SAMR1 control mice, but not in female mice, whereas H3K27me3 was reduced overall in SAMP8 mice. Moreover, the levels of HDAC2 and *JmjD3* were increased, whereas the levels of HDAC4 and *Dnmt3a* were reduced in SAMP8 mice compared to SAMR1. In addition, levels of HDAC1 were reduced, whereas *Utx* and *Jmjd3* were selectively increased in females compared to males. Although our results are preliminary, they suggest that epigenetic mechanisms in the dorsal hippocampus are differentially regulated in male and female SAMP8 mice.

## 1. Introduction

Alzheimer’s disease (AD) is the most common form of dementia, characterized by progressive memory loss and cognitive decline [1]. As aging is the most important risk factor for the development of age-related cognitive impairment and neurodegenerative disorders, the prevalence of AD increases with human life expectancy, reaching 30–40% in people over 85 years of age, and is expected to increase to over 91 million worldwide by 2050 [2]. Depending on the age at which the disease occurs, AD can be divided into early-onset AD and late-onset AD. The sporadic late-onset AD occurs later in life (after age 65) and is far more common, affecting 95% of AD patients, while the familiar early-onset AD occurs early in life (before age 65) and is less common, affecting only 5% of AD patients [3,4]. Although the etiology of AD is still not well understood, it involves a complex interaction between genetic, environmental, and epigenetic factors. Epigenetic mechanisms regulate chromatin structure and gene expression without altering the DNA sequence. They are modifiable, transmissible, and strongly influenced by environmental factors [5,6]. There is increasing evidence that epigenetic mechanisms, including post-translational histone modifications and DNA methylation, play a key role in learning and memory processes, and alterations in these mechanisms have been linked to AD pathogenesis [7,8].

Different enzymes are involved in the regulation of epigenetic marks, classified as writers, erasers, and readers [9,10,11,12]. The writers are responsible for adding various chemical modifications to DNA and histones and include DNA methyltransferases (DNMTs), histone acetyltransferases (HATs), and histone methyltransferases (HMTs) [9,10,11,12]. Erasers are responsible for removing these chemical marks and include histone deacetylases (HDACs), histone demethylases, and proteins involved in DNA demethylation [9,10,11,12]. Readers are proteins that recognize and bind to specific epigenetic marks. They convert them into functional outcomes by recruiting other proteins or complexes that include chromatin-associated proteins and DNA-binding proteins [9,10,11,12].

Histone acetylation at lysine residues, modulated by the opposing activities of HATs and HDACs, is generally associated with active gene transcription and open chromatin conformations, making DNA more accessible to the transcription machinery [13,14]. Conversely, histone methylation at lysine, arginine, or histidine residues, which is regulated by HMTs and histone demethylases, can cause both activation and repression of gene transcription, depending on which residues are methylated and the extent of the methylation [14]. Finally, DNA methylation is associated with gene repression and is regulated by DNMTs and ten eleven translocation (TET) methylcytosine dioxygenase [15].

Numerous aspects of neuronal function and development are regulated by epigenetic factors and there is growing evidence that chromatin organization plays an important role in brain aging, cognitive decline, and neurodegeneration [16,17]. Remarkably, nuclear Tau has recently been reported to play a physiological function in the assembly and maintenance of heterochromatin by regulating epigenetic mechanisms. Heterochromatin structure and epigenetic marks are altered by pathological hyperphosphorylated Tau, a feature of AD [18,19,20]. Therefore, epigenetic changes associated with brain aging may be important targets to halt cognitive decline and neurodegeneration.

Animal models are useful to better understand the pathogenesis of diseases and to develop new therapeutic approaches. Most of the animal models of AD are transgenic mice showing cognitive impairment and neurodegeneration early in life and, therefore, essentially recapitulate the familiar early-onset AD [21,22]. The senescence-accelerated mouse prone 8 (SAMP8) and its control (senescence-accelerated mouse resistant, SAMR1, characterized by normal aging) are strains spontaneously developed by breeding from the AKR/J colony [23]. SAMP8 mice are widely used as a model of physical and mental aging and have been proposed as a mouse model of neurodegeneration to study late-onset AD [23,24]. Indeed, the SAMP8 mouse shows learning and memory deficits along with other AD-like traits, including Tau hyperphosphorylation, beta amyloid accumulation, altered gene expression, oxidative stress, gliosis, and neuronal loss [25,26]. Altered epigenetic regulations have been described in SAMP8 mice compared to SAMR1 mice [27,28,29,30,31]; however, it is not known whether epigenetic changes are different between sexes.

The aim of this work was to characterize the level of posttranslational histone modifications and epigenetic regulatory enzymes in the dorsal hippocampus, a brain region involved in learning and memory, of 9-month-old male and female SAMP8 mice compared to male and female SAMR1 control mice.

## 2. Results

### 2.1. Markers of Neurodegeneration Are Altered in 9-Month-Old SAMP8 Mice

First, we measured the levels of some molecular effectors that have been extensively reported to be altered in neurodegenerative diseases including AD, such as brain-derived neurotrophic factor (*Bdnf*) [32], glial fibrillary acid protein (*Gfap*) [33,34], ionized calcium-binding adapter molecule 1 (*Iba1*) [34,35], and Site APP-cleaving enzyme 1 (BACE1) [36], in 9-month-old SAMP8 and SAMR1 mice.

**Figure 1:**
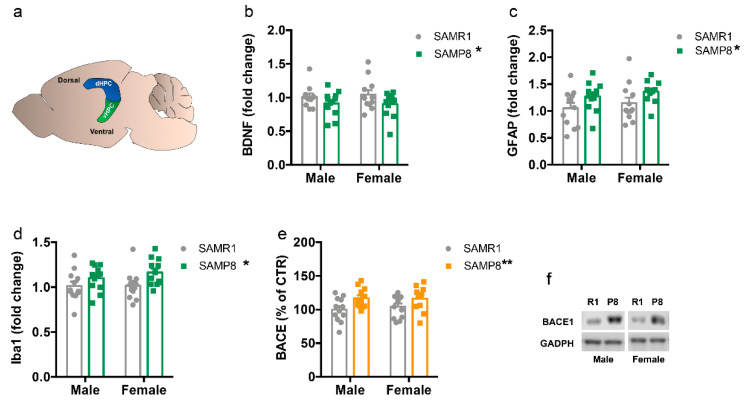
Levels of *Bdnf*, *Gfap, Iba1*, and BACE in the dorsal hippocampus of male and female SAMP8 mice compared to SAMR1. Schematic representations of the dorsal and ventral hippocampal region in the mouse brain (**a**). mRNA levels of *Bdnf* (**b**), *Gfap* (**c**) and *Iba1* (**d**) in the dorsal hippocampus of 9-month-old male and female SAMR1 and SAMP8 mice were measured by qPCR. Densitometric analysis of BACE1 protein levels were determined as the ratio of BACE1/GAPDH (**e**). Representative western blot images (**f**). R1: SAMR1, P8: SAMP8. Data are presented as mean ± SEM. N = 11–12 mice/group. Two-way ANOVA followed by Tukey’s post hoc analysis. * *p* < 0.05; ** *p* < 0.01.

The total amount of *Bdnf* mRNA was decreased in SAMP8 compared to SAMR1 (F_(1,43)_ = 4.629, *p* = 0.0371), and this reduction was similar in male and female mice (sex: F_(1,43)_ = 4.629, *p* = 0.8443; interaction: F_(1,43)_ = 0.149, *p* = 0.701) (Figure 1b,f). Conversely, the overall mRNA levels of *Gfap* (F_(1,44)_ = 76.354, *p* = 0.015) and Iba1 (F_(1,43)_ = 7.48, *p* = 0.011), and protein levels of BACE1 (F_(1,43)_ = 8.618, *p* = 0.0053) were higher in the dorsal hippocampus of SAMP8 mice compared to SAMR1 mice, regardless of sex (Figure 1c–f).

### 2.2. Histone Post-Translational Modifications Are Altered in the Dorsal Hippocampus of 9-Month-Old SAMP8 Mice

Regarding histone modifications, we analyzed global acetylation of histone 3 (H3Ac) and histone 4 (H4Ac) as well as trimethylation of H3 at lysine 27 (H3K27me3), which have been reported to be altered in AD and neurodegeneration [37,38,39,40].

**Figure 2:**
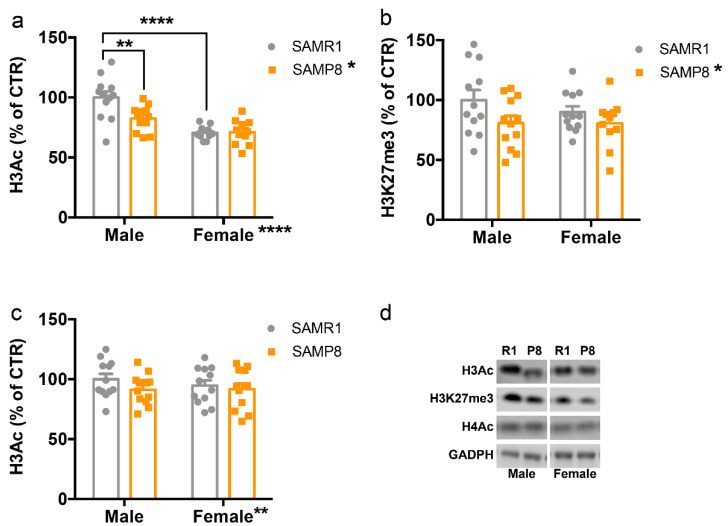
Histone post-translational modifications in the dorsal hippocampus of male and female SAMP8 mice compared to SAMR1. Levels of acetylated histone H3 (H3Ac) (**a**), tri-methylated histone H3 (H3K27me3) (**b**), and acetylated histone H3 (H4Ac) (**c**) in the dorsal hippocampus of 9-month-old male and female SAMR1 and SAMP8 mice were measured by western blot. (**d**) representative western blot images. R1: SAMR1, P8: SAMP8. Data are presented as mean ± SEM. N = 11–12 mice/group. Two-way ANOVA followed by Tukey’s post hoc analysis. * *p* < 0.05; ** *p* < 0.01; **** *p* < 0.0001.

Analysis of H3Ac levels revealed a main effect of genotype (F_(1,43)_ = 6.612, *p* = 0.0183), sex (F_(1,43)_ = 38.34, *p* < 0.0001), and sex x genotype interaction (F_(1,43)_ = 7.38, *p* = 0.0131) (Figure 2a). Interestingly, the Tukey’s post hoc analysis showed that the reduction of H3Ac in SAMP8 mice compared to SAMR1 was statistically significant only in males (*p* < 0.01), but not in females (*p* > 0.05) (Figure 2a). Conversely, H3K27me3 levels were reduced in SAMP8 mice compared to SAMR1 (F_(1,43)_ = 9.78, *p* = 0.0341) regardless of sex (Figure 2b), whereas H4Ac levels were similar in all mouse groups (Figure 2c).

### 2.3. HDACs Protein Levels Are Altered in the Dorsal Hippocampus of 9-Month-Old SAMP8 Mice

**Figure 3:**
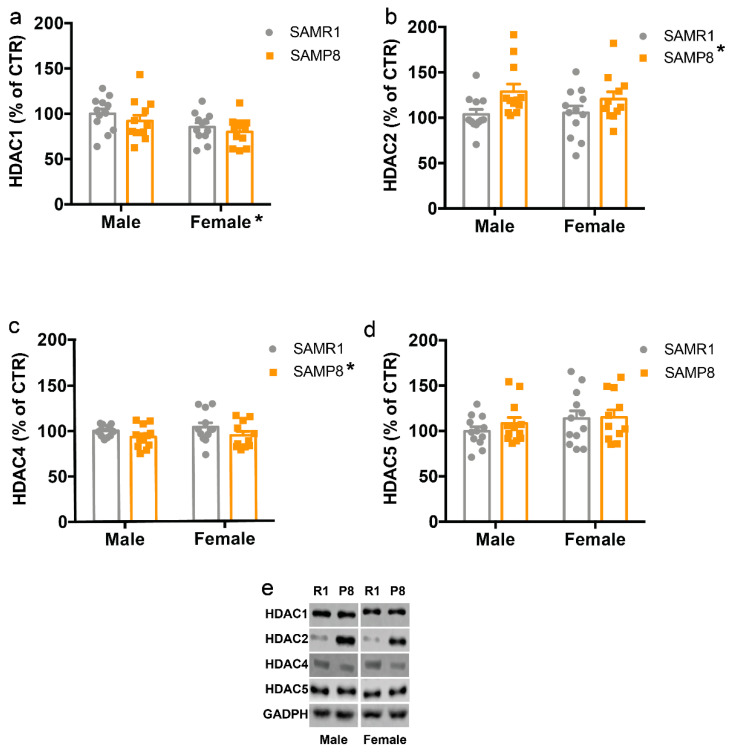
HDACs protein levels in the dorsal hippocampus of male and female SAMP8 mice compared to SAMR1. Levels of HADCs in the dorsal hippocampus of 9-month-old male and female SAMR1 and SAMP8 mice were measured by western blot analysis. (**a**–**d**) Densitometric analysis of HADC1 (**a**); HADC2 (**b**); HADC3 (**c**); HADC4 (**d**). (**e**) representative western blot images. R1: SAMR1, P8: SAMP8. Data are presented as mean ± SEM. N = 11–12 mice/group. Two-way ANOVA followed by Tukey’s post hoc analysis. * *p* < 0.05.

In search of possible mechanisms behind the reduction of H3Ac in SAMP8 mice, we then examined the protein levels of some histone deacetylases (HDACs). We found that HDAC1 levels were reduced in females compared to males (F_(1,43)_ = 12.10, *p* = 0.0173) with no effect of genotype (Figure 3A), HDAC2 levels were increased in SAMP8 mice compared to SAMR1 mice (F_(1,43)_ = 10.09, *p* = 0.031) with no effect of sex (Figure 3B), while HDAC4 amount was decreased in SAMP8 mice compared to SAMR1 mice (F_(1,43)_ = 9.16, *p* = 0.042) with no effect of sex (Figure 3C). No significant differences were observed for HDAC5 (Figure 3D).

### 2.4. Histone Demethylase Levels Are Increased in the Dorsal Hippocampus of 9-Month-Old SAMP8 Mice

**Figure 4:**
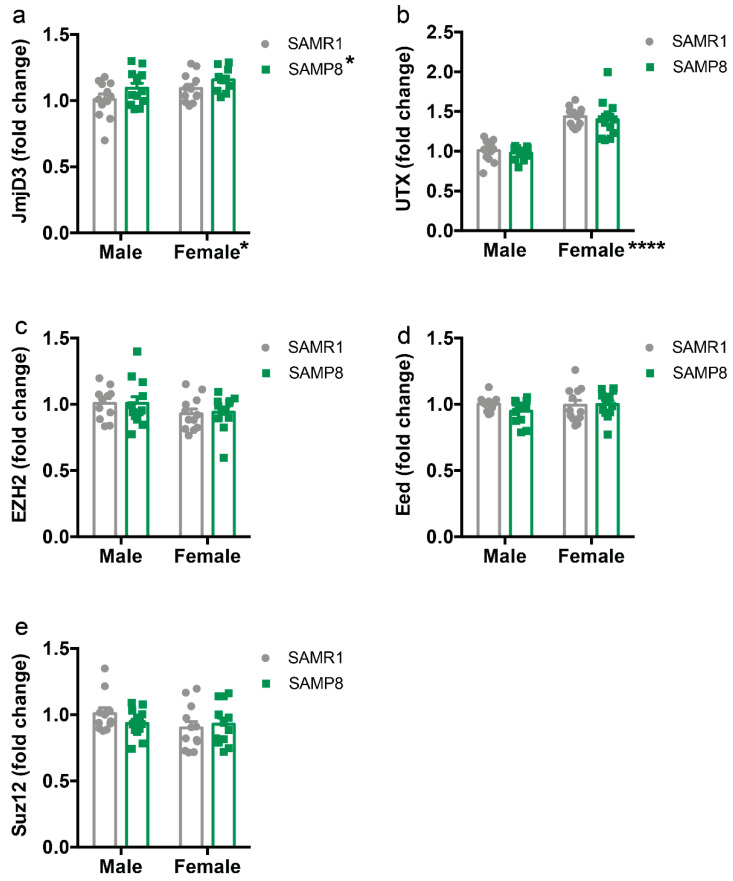
Histone demethylase levels in the dorsal hippocampus of male and female SAMP8 mice compared to SAMR1. mRNA levels of *Jmjd3* (**a**), *Utx* (**b**), *Ezh2* (**c**), *Eed* (**d**), and *Suz12* (**e**) in the dorsal hippocampus of 9-month-old male and female SAMR1 and SAMP8 mice were measured by qPCR. Data are presented as mean ± SEM. N = 11–12 mice/group. Two-way ANOVA followed by Tukey’s post hoc analysis. * *p* < 0.05; **** *p* < 0.0001.

After detecting a global decrease in H3K27me3 levels in SAMP8 mice, we examined the levels of specific methylases, and demethylases involved in the regulation of H3K27 methylation. Regarding histone demethylases, the two-way ANOVA revealed a main effect for genotype (F_(1,43)_ = 9.16, *p* = 0.042) and sex (F_(1,43)_ = 9.16, *p* = 0.042) for Jumonji domain-containing protein-3 (*Jmjd3*), with higher levels in females compared to males, and in SAMP8 compared to SAMR1 (Figure 4a). In addition, *Utx* levels were higher in female mice than in males (F_(1,43)_ = 9.16, *p* = 0.042) with no effect of genotype (Figure 4b). Conversely, the levels of the core components of histone methyltransferase Polycomb Repressive Complex 2 (PRC2), including enhancer of zeste homolog 2 (*Ezh2*), embryonic ectoderm development (*Eed*), and PRC2 subunit (*Suz12*), which catalyzes trimethylation of H3K27, were comparable in all groups (Figure 4c–e).

### 2.5. Dnmt3a Levels Are Reduced in the Dorsal Hippocampus of 9-Month-Old SAMP8 Mice

**Figure 5:**
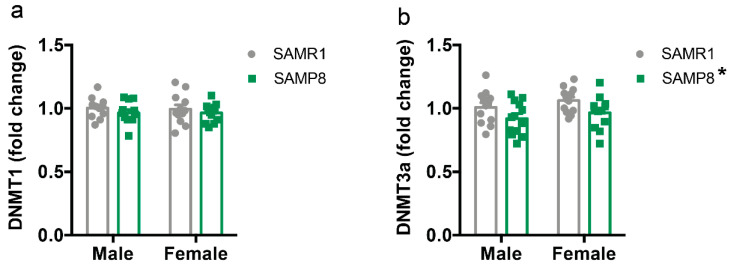
Levels of DNA methyltransferases in the dorsal hippocampus of male and female SAMP8 mice compared to SAMR1. mRNA levels of *Dnmt1* (**a**) and *Dnmt3a* (**b**) in the dorsal hippocampus of 9-month-old male and female SAMR1 and SAMP8 mice were measured by qPCR. Data are presented as mean ± SEM. N = 11–12 mice/group. Two-way ANOVA followed by Tukey’s post hoc analysis. * *p* < 0.05.

Finally, we measured the mRNA expression levels of DNA methyltransferases Dnmt1 and Dnmt3a in the dorsal hippocampus. The levels of Dnmt1 were unaffected by genotype or sex (Figure 5a). In contrast, global levels of Dnmt3a were lower in SAMP8 mice than in SAMR1 mice (F_(1,43)_ = 9.16, *p* = 0.042) with no effect of sex (Figure 5b).

## 3. Discussion

In the present study, we aimed to investigate the epigenetic landscape in the dorsal hippocampus, a brain region involved in learning and memory, in both male and female 9-month-old SAMP8 mice, as female mice are an underrepresented model of the disease and females are more likely to develop AD [41,42,43]. We found a selective reduction of H3Ac in male SAMP8 mice, but not in females, and a general reduction of H3K27me3 in SAMP8 mice. These changes were accompanied by an increase in protein levels of HDAC2 and mRNA of *Jmjd3* in SAMP8 mice compared to SAMR1. In contrast, HDAC4 and *Dnmt3a* were reduced in male and female SAMP8 mice compared to SAMR1, whereas females showed a reduction of HDAC1 and an increase of *Utx* levels.

Previous studies showed that SAMP8 mice exhibit several AD pathological features in the hippocampus and other brain regions, including increased amyloid-β (Aβ) protein precursor, increased Aβ protein, amyloid-like plaque deposits, hyperphosphorylation of Tau protein, decreased dendritic spine density, gliosis, reduced neurotrophic factors, and neuronal loss [25,44,45]. Remarkably, Tau hyperphosphorylation and Aβ deposition in the hippocampus were detected as early as 3 and 6 months of age, respectively, and increase with age [25,44,45]. All of these changes mimic the pathologies of AD patients, making SAMP8 mice a valuable model to study AD. Consistent with these findings, we found an increase in hippocampal BACE1, *Gfap*, and *Iba1* levels with a concomitant decrease in *Bdnf* in 9-month-old SAMP8 mice compared to SAMR1 mice.

Dysregulations of epigenetic mechanisms have been linked to the pathophysiology of several neurodegenerative diseases, including AD. For example, a decrease in histone acetylation in the temporal lobe was observed in AD patients [46]. Furthermore, a specific decrease in H4K16Ac was observed in the cortex of AD patients compared to healthy individuals [47]. On the other hand, a significant enrichment of H3K27Ac was detected in the cortex of AD patients in genes involved in the progression of AD [48]. Similar histone acetylation reduction was also reported in AD moused models [49,50,51]. Here, we found a selective reduction of H3Ac in male SAMP8 mice compared to SAMR1 mice, but not in female mice. However, H3Ac levels were already reduced in female mice compared to male mice, which may have compromised the ability to induce a further reduction. Moreover, the lack of a significant difference between SAMP8 and SAMR1 female mice at 9 months of age is consistent with a previous study that found a reduction of H3Ac in the whole hippocampus of 2-month-old but not 9-month-old female SAMP8 mice compared to SAMR1 [27]. In future studies, it would be interesting to analyze whether the reduction of H3Ac in male SAMP8 mice may occur also at different ages and what the epigenetic changes are across the lifespan in both male and female mice.

In search of possible mechanisms underlying the decrease of H3Ac in male SAMP8 mice, we analyzed the levels of some HDACs and found a selective increase of HDAC2 in SAMP8 mice and a decrease of HDAC4 in females in the dorsal hippocampus. Consistent with our data, HDAC2 was previously reported to be increased in the hippocampus and prefrontal cortex of both AD mouse models and AD patients [52]. In addition, HDAC2 levels have been reported to be enriched at the promoters of genes involved in learning, memory, and synaptic plasticity. This leads to a decrease in histone acetylation at the same promoters and a decrease in the expression of the corresponding genes [52]. As a result, elevated HDAC2 levels may lead to decreased synaptic function, a pathogenic feature of AD, which has been extensively studied [53,54]. Conversely, HDAC2 deficiency resulted in increased synapse number and memory facilitation, and the same occurs with chronic treatment with HDAC inhibitors in mice [54].

In contrast to the role of HDAC2, the expression of HDAC4 has been associated with neuroprotective effects. Indeed, selective loss of HDAC4 in the brain leads to impairments in hippocampus-dependent learning and memory as well as in long-term synaptic plasticity [55]. Moreover, overexpression of cytoplasmic HDAC4 rescues spine density and synaptic transmission in an AD mouse model [56]. Altogether, these results suggest that different HDACs may play opposing roles in the pathogenesis of AD. Another important epigenetic modification is histone methylation. Here, we found an overall reduction of H3K27me3 in SAMP8 mice compared to the SAMR1 control group. H3K27 is regulated by the Polycomb protein complex, which consists of the EZH2, Suz12, and EED proteins [57], whereas its demethylation is regulated by the JMJD3 and UTX demethylases [58,59]. Consistent with the decrease of H3K27me3, we found an increase of *Jmjd3* in the dorsal hippocampus of SAMP8 mice compared to SAMR1. Remarkably, alterations in H3K27me3 levels have recently been linked to various neurodegenerative diseases [37,60,61]. In particular, it has been suggested that a reduction in H3K27me3 levels leads to the de-repression of selected genes that are normally repressed in neuronal cells, which in turn promotes progressive and lethal neurodegeneration [37]. Therefore, we may speculate that reduced H3K27me3 levels may contribute to the enhanced neuronal cell death previously described in SAMP8 mice [62,63].

Although our results showed sex-specific difference in the expression of some epigenetic markers, more studies are required to understand whether these changes could be associated to specific behavioral phenotypes and different susceptibility to AD-like pathogenesis in SAMP8 male and female mice.

## 4. Materials and Methods

### 4.1. Animals

Male and female SAMP8 and SAMR1 mice were purchased from ENVIGO RMS RSL (San Pietro al Natisone, UD, Italy). Mice were housed under standard conditions (20–22 °C, 12 h light/dark cycle, light on at 7 a.m.), with water and food ad libitum. Animal husbandry and experimental procedures were performed in accordance with European Community Council Directive 2010/63/UE and approved by the Italian legislation on animal experimentation (Decreto Legislativo 26/2014, authorization N 951/2018-PR). All efforts were made to minimize the stress on mice and reduce the number of animals used in this study.

### 4.2. Western Blot Analysis

Western blot analysis was performed as previously described [64]. Dissected dorsal hippocampi (Bregma from −0.90 to −2.40) were homogenized in ice-cold RIPA buffer (15 mM NaCl, 5 mM Tris HCl, pH 7.4, 5 mM EDTA, 1% Triton X-100, 1% sodium deoxycholate, and 0.1% SDS) with Protease Inhibitor Cocktail (Sigma-Aldrich, Milan, Italy). Protein concentration was analyzed by Quantum Bicinchoninic Protein Assay (Euroclone, Pero MI, Italy), and 15–30 μg of proteins were separated on SDS-PAGE gels and blotted onto a polyvinylidene difluoride membrane (GE Healthcare Life Sciences, Milan, Italy). The blotted membranes were saturated in 5% milk in Tris Buffer Saline-Tween 20 (TBS-T) and treated with anti-BACE1 (1:1000, Abcam, Cambridge, UK); anti-HDAC1 (1:1000 Cell Signalling Technology, Danvers, MA, USA); anti-HDAC2 (1:1000 Cell Signalling); anti-HDAC4 (1:1000 Cell Signalling); anti-HDAC5 (1:1000 Santa Cruz Biotechnology, Dallas, TX, USA); or anti-GAPDH (1:2500, Merck-Millipore, Milano, Italy) antibodies. Membranes were washed several times with TBS-T and then incubated with the appropriate peroxidase-conjugated secondary antibody (1:5000, Sigma-Aldrich) or fluorescent IRDye secondary antibody (1:5000, LI-COR Biosciences, Lincoln, NE, USA). Peroxidase immunoreactivity bands were detected by chemiluminescence using the ECL detection system (Bio-Rad Laboratories, Milan, Italy). Chemiluminescence and fluorescence membrane signals were scanned and quantified using an Odyssey LI-COR scanner (LI-COR Biosciences)

### 4.3. RNA Isolation and Reverse Transcription

RNA isolation and reverse transcription were performed as described previously [65]. Briefly, total RNA was extracted from dorsal hippocampi using the Direct-zol^TM^ RNA MiniPrep (Zymo Research, Irvine, CA, USA) according to the manufacturer’s instructions [66]. cDNA was synthesized using the iScript kit (Bio-Rad Laboratories), which contains a blend of oligo(dT) and random hexamer primers in the iScript Reaction Mix, according to the manufacturer’s instructions. The specificity of PCR products was assessed by melting curve analysis.

### 4.4. Quantitative PCR

Quantitative real-time PCR (qPCR) analysis was performed on a CFX Connect Real-Time System (Bio-Rad Laboratories) using iTaq Universal SYBR Green Supermix (Bio-Rad Laboratories). The primer sequences used are listed in Table 1. The qPCR conditions were 2.5 min at 95 °C, 40 cycles of 15 s at 95 °C, and 30 sec at 60 °C. Relative mRNA expression was calculated by the comparative CT method using the formula 2^−∆∆CT^. GAPDH was used as endogenous control gene. Relative mRNA levels were expressed as fold changes. The specificity of PCR products was assessed by melting curve analysis.

### 4.5. Statistical Analysis

Statistical analysis of the data was carried out using GraphPad Prism9 (GraphPad Software Inc., Boston, MA, USA). Results are presented as mean ± standard error of the mean (SEM). All the data were analyzed using a two-way ANOVA followed by Tukey’s post hoc multiple comparison test, when appropriate.

## 5. Conclusions

In summary, we have reported here for the first time that epigenetic mechanisms in the dorsal hippocampus are differentially altered in male and female SAMP8 mice, further supporting the possible role of epigenetic mechanisms in sex difference in age-related brain diseases, including AD.

A major limitation of this study is that it is preliminary, because we analyzed only global histone changes and not specific histone changes at the promoters of genes involved in AD. Future studies are, therefore, needed to better characterize and, more importantly, modulate epigenetic markers at the level of promoters of genes involved in AD to unravel the role of epigenetic markers in AD-like pathogenesis in SAMP8 mice. In addition, immunofluorescence studies should also be performed to identify the hippocampal subregions and the specific neuronal and/or glial cell types in which these epigenetic changes occur.

Another limitation of the study is that we did not perform studies to correlate these epigenetic changes with behavioral analyses. Importantly, to our knowledge, it is not known whether male and female SAMP8 mice exhibit differences in learning and memory because male and female mice have always been studied separately in previous studies. Therefore, future studies are needed to understand whether sex differences in epigenetic changes are associated with specific behavioral changes. Moreover, in our study we analyzed only the dorsal hippocampus, but, in future studies, it would be interesting to examine other brain regions at different time points to determine whether epigenetic markers in different brain regions are affected by age and sex.

Although preliminary, our results suggest sex differences in the epigenetic profile of SAMP8 mice.

## Figures and Tables

**Table 1:** List of primers.

Gene	Primer Forward	Primer Reverse	Size bp
*Bdnf*	TCGTTCCTTTCGAGTTAGCC	TTGGTAAACGGCACAAAAC	97
*Gfap*	GGCAGAAGCTCCAAGATGAAAC	TCCAGCGATTCAACCTTTCTCT	127
*Iba1*	AGAGATCTGCCATCTTGAGAAT	TGACTCTGGCTCACGACTGTT	195
*Utx*	TGCCTTACCTGCAGCGAAA	ACCTTTGTGAAGCCCCTGAGT	119
*Jmjd3*	TGGTTCACTTCGGCTCAACTTAG	TCTCGTCTGAGGGCTGCTGTA	110
*Ezh2*	CAACCCTGTGACCATCCACG	CGACATCCAGGAAAGCGGTT	122
*Eed*	CGGGAGACGAAAATGACGAT	CTTTTCCTTCCTGGTGCATTTG	100
*Suz12*	CCGGTGAAGAAGCCGAAAAA	TATTGGTGCGATAGATTTCGAGTT	119
*Dnmt1*	GGACACAGGTGCCCGCGA	ATGAACCCCAGATGTTGACCA	136
*Dnmt3a* *Gapdh*	AGATCATGTACGTCGGGGAC CGTGCCGCCTGGAGAAACC	CAATCACCAGGTCGAATGGG CGTGCCGCCTGGAGAAACC	78 144

## Data Availability

The data that support the findings of this study are available from the corresponding author upon reasonable request.
